# Effects of germination season on life history traits and on transgenerational plasticity in seed dormancy in a cold desert annual

**DOI:** 10.1038/srep25076

**Published:** 2016-04-27

**Authors:** Juan J. Lu, Dun Y. Tan, Carol C. Baskin, Jerry M. Baskin

**Affiliations:** 1 Xinjiang Key Laboratory of Soil and Plant Ecological Processes, College of Grassland and Environment Sciences, Xinjiang Agricultural University, Urumqi 830052, China; 2 Department of Biology, University of Kentucky, Lexington, KY 40506, USA; 3 Department of Plant and Soil Sciences, University of Kentucky, Lexington, KY 40546, USA

## Abstract

The maternal environment can influence the intensity of seed dormancy and thus seasonal germination timing and post-germination life history traits. We tested the hypotheses that germination season influences phenotypic expression of post-germination life history traits in the cold desert annual *Isatis violascens* and that plants from autumn- and spring-germinating seeds produce different proportions of seeds with nondeep and intermediate physiological dormancy (PD). Seeds were sown in summer and flexibility in various life history traits determined for plants that germinated in autumn and in spring. A higher percentage of spring- than of autumn-germinating plants survived the seedling stage, and all surviving plants reproduced. Number of silicles increased with plant size (autumn- > spring-germinating plants), whereas percent dry mass allocated to reproduction was higher in spring- than in autumn-germinating plants. Autumn-germinating plants produced proportionally more seeds with intermediate PD than spring-germinating plants, while spring-germinating plants produced proportionally more seeds with nondeep PD than autumn-germinating plants. Flexibility throughout the life history and transgenerational plasticity in seed dormancy are adaptations of *I. violascens* to its desert habitat. Our study is the first to demonstrate that autumn- and spring-germinating plants in a species population differ in proportion of seeds produced with different levels of PD.

Germination is highly responsive to the immediate environment of the seeds^1^ as well as to that experienced by the mother plant during seed development which can influence the intensity of dormancy^2^,^3^,^4^. Consequently, if environments change due to habitat alteration or global warming germination behavior is likely to change as a direct and immediate response. For example, in species that typically can germinate in early autumn protracted summer drought conditions could cause germination to be delayed until later in the autumn or even until the following spring. Such a delay not only may have direct fitness consequences but also affect other aspects of the plants’ life history^5^,^6^,^7^,^8^,^9^,^10^,^11^,^12^. Germination timing also may be altered by evolutionary responses to selection on germination^9^,^10^. Therefore, characterizing how the timing of seed germination (F_1_) influences phenotypic expression, fitness^10^,^11^,^12^,^13^,^14^ and possibly dormancy-breaking/germination requirements of the resulting seeds (F_2_) provides information on the manner in which plants might be affected by environmentally induced or evolutionary changes in their germination behavior.

For summer and winter annuals, germination of seeds in early spring and early autumn, respectively, allows plants to have the full growing season for growth and seed set, thereby attaining a large size and producing many seeds. However, some species can behave as both winter and short-lived summer annuals (ephemerals) and thus seeds germinate in autumn and in spring with seed set being completed in late spring/early summer for both cohorts; these species are called facultative winter annuals. Many plant species in the Gurbantuggut Desert in the Junggar Basin of Xinjiang Uyghur Autonomous Region of NW China typically behave, or have the potential to behave, as facultative winter annuals^11^,^12^,^15^,^16^,^17^,^18^,^19^.

If the soil is sufficiently moist to promote germination in autumn in this cold desert, seeds germinate and surviving plants have a winter annual life cycle. If the soil is too dry for seeds to germinate in autumn, germination is delayed until spring, at which time the soil is moist due to rain and/or melting snow^20^,^21^, and seeds germinate and plants behave as ephemerals^11^,^12^,^16^,^17^. In years with a reasonable amount of rainfall in autumn, a portion of the seed population of an annual species germinates in autumn and another portion in spring^11^,^12^,^16^,^17^, i.e. the species behaves as a facultative winter annual.

Zhou *et al.*^22^ recently have shown that there is another mechanism by which seeds of annual plants in the cold deserts of Central Asia can time germination. Dispersal units (i.e. one-seeded indehiscent fruits, hereafter seeds) of *Isatis violascens* Bunge (Brassicaceae) have either nondeep or intermediate physiological dormancy (PD). These two levels of PD allow *I. violascens* to have considerable flexibility in its life cycle phenology. Seeds with nondeep PD afterripen (come out of dormancy) during summer and can germinate in autumn if the soil is moist. If they fail to germinate in autumn they can do so in spring. Seeds with intermediate PD germinate in spring after they have received a period of afterripening during summer that is followed by cold stratification during winter. Thus, *I. violascens* can behave as a facultative winter annual by seeds with nondeep PD germinating in autumn and in spring and those with intermediate PD germinating only in spring. However, if soil moisture is insufficient for germination in autumn, some seeds with nondeep PD can germinate in spring. We tested two hypotheses related to the life history of *I. violascens*. Firstly, since *I. violascens* can behave as a facultative winter annual we hypothesized that germination timing (autumn versus spring) would influence the phenotypic expression of post-germination life history traits including seed production, a component of fitness^23^. In *Arabidopsis thaliana*, germination timing not only influences plant fitness but also seed germination traits of the offspring^24^,^25^. To test our germination timing hypothesis, we compared phenology, growth and morphology, survival, biomass accumulation and allocation and silicle and seed production of plants from autumn- versus spring-germinating seeds of *I. violascens*. Secondly, it is well known that the environment in which mother plants are grown can influence dormancy and germination characteristics of the seeds produced, i.e. transgenerational plasticity^1^,^26^,^27^. Thus, we hypothesized that plants from autumn- and spring-germinating seeds (F_1_) would produce different proportions of seeds with nondeep and intermediate PD. To test this hypothesis, seeds (F_2_) produced at the same time from plants that germinated in autumn and in spring were subjected to dormancy-breaking treatments and germination tests to determine the proportion of seeds with nondeep and intermediate PD.

## Results

### Germination season causes flexibility of life history traits

Germination season affected key stages of the life cycle, except for flowering date and fruiting date (Table 1; Supplementary Table S1).

#### Phenology

Plants from seeds that germinated in autumn behaved as winter annuals, and those from seeds that germinated in spring behaved as spring ephemerals. The interval from seedling emergence to flowering in autumn-germinating plants (209 days) was significantly longer than that in spring-germinating plants (39 days) (Supplementary Table S1). Flowering period was positively correlated with plant size (height) at maturity (Supplementary Table S2). Autumn-germinating plants had a longer post-germination life span than spring-germinating plants, but the proportion of the reproductive period for the whole post-germination life span of autumn-germinating plants was significantly shorter than the proportions for spring-germinating plants (Supplementary Table S2).

#### Survivorship

Sixteen plants (52%) from the 31 seeds that germinated in autumn 2013 were alive and in the rosette stage in spring 2014, and 55 plants (89%) from the 62 seeds that germinated in early spring 2014 were alive and in the 4-leaf stage in late spring 2014. All 16 autumn-germinating plants and all 55 spring-germinating plants reproduced.

#### Morphological characters

Height of autumn-germinating plants was significantly greater than that of spring-germinating plants (Supplementary Table S1).

#### Silicle production

Spring-germinating plants produced significantly fewer infructescences and silicles per individual than autumn-germinating plants (Supplementary Table S1). Plant height was positively correlated with number of silicles (Supplementary Table S2).

#### Dry mass accumulation and allocation

**Figure 1:**
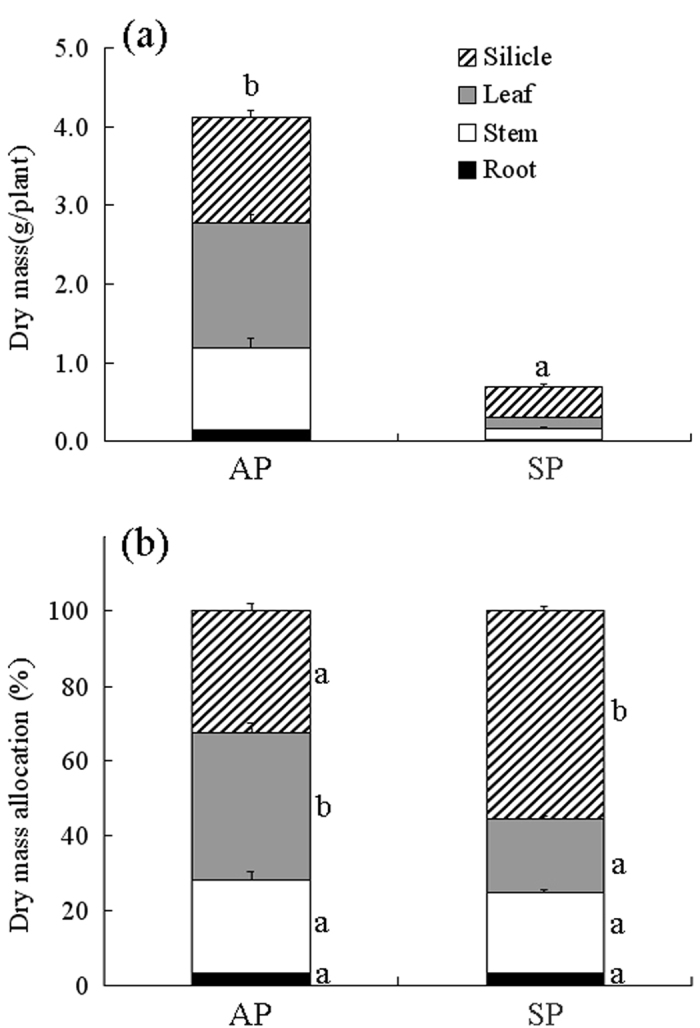
Effect of germination season on (a) dry mass accumulation and (b) allocation (mean + 1 s.e.) in *Isatis violascens*. AP, autumn-germinating plants; SP, spring-germinating plants. Bars with different letters for total dry mass (**a**) or for portions of dry mass allocation (**b**) indicate significant difference in multiple range comparison (Tukey’s HSD, P < 0.05).

Total dry mass of autumn-and spring-germinating plants was 4.12 and 0.70 g plant^−1^, respectively (Table 1; Fig. 1a), and dry mass of reproductive organs was 1.34 and 0.40 g plant^−1^, respectively. Dry mass of reproductive organs was significantly positively correlated with plant height (r = 0.72, P < 0.001).

Allocation to vegetative organs was higher in autumn-germinating plants than in spring-germinating plants, with the opposite result for dry mass allocation to reproductive organs (Fig. 1b). Allocation of dry mass to reproduction was 32.6% and 55.7% in autumn- and spring-germinating plants, respectively (Table 1; Fig. 1b). Allocation of dry mass to reproduction was significantly negatively correlated with plant height (r = −0.57, P < 0.001). The high proportion of dry mass allocated to reproduction resulted in a relatively small amount allocated to vegetative organs.

### Seed dormancy exhibits transgenerational plasticity

#### Presence of nondeep PD

**Figure 2:**
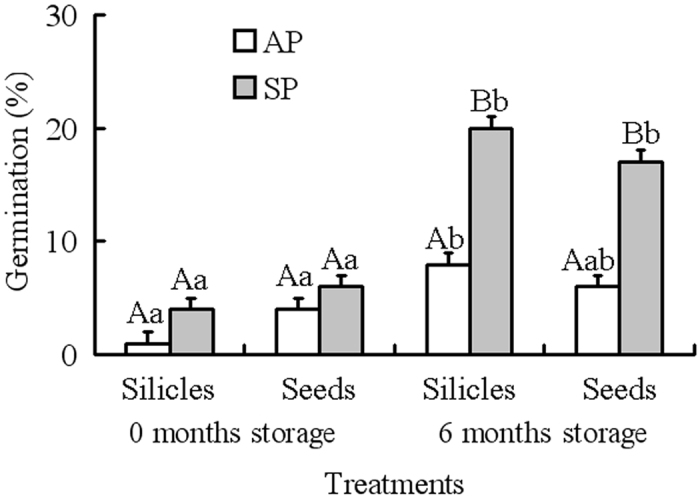
Germination of 0- and 6-month-old silicles and seeds from autumn- and spring-germinating plants (mean + 1 s.e.) of *Isatis violascens* incubated at 5/2 °C in darkness. AP, autumn-germinating plants; SP, spring-germinating plants. Different lowercase letters indicate significant differences (P < 0.05) among the different storage times (0 and 6 mo) and different pericarp treatments (intact fruit vs. isolated seed) for autumn- and spring-germinating plants and different uppercase letters significant differences between autumn- and spring-germinating plants at the same storage time and the same pericarp treatment.

GLM analysis showed that germination was significantly affected by germination time of the mother plants and storage time (Table 2). However, the effect of treatment (i.e. intact silicles and isolated seeds) and any interactions was not significant (Table 2). At storage time zero, the highest germination was less than 6% for intact silicles and isolated seeds from autumn- and from spring-germinating plants (Fig. 2). After 6 months of dry storage, 20% and 17% of seeds in silicles and isolated seeds from spring-germinating plants germinated, respectively, but only 8% and 6% of those from autumn-germinating plants germinated, respectively (Fig. 2).

#### Presence of intermediate PD

**Figure 3:**
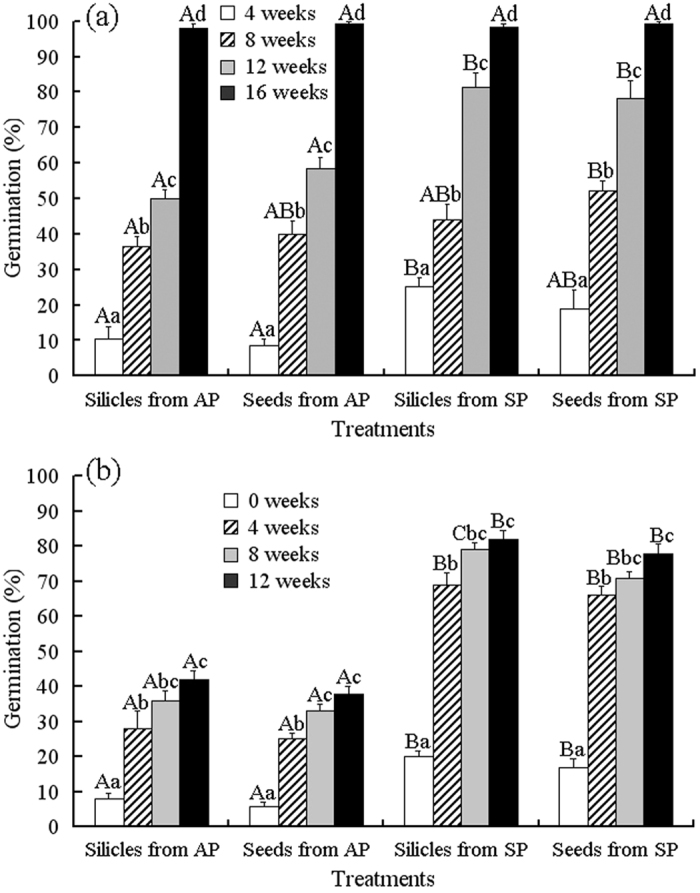
Effect of cold stratification on germination (mean + 1 s.e.) of 6-month-old dry-stored silicles and seeds from autumn- and spring-germinating plants of *Isatis violascens* (a) during cold stratification at 4 °C for 4, 8, 12 and 16 weeks and (b) during incubated at 5/2 °C in darkness after cold stratification for 0, 4, 8 and 12 weeks. Different uppercase letters indicate significant differences (P < 0.05) among different treatment at the same cold stratification time and different lowercase letters significant differences among different cold stratification times within the same treatment. AP, autumn-germinating plants; SP, spring-germinating plants.

GLM analysis showed that the effect of germination time of mother plants and cold stratification time were highly significant, but effect of treatment (i.e. intact silicles and isolated seeds) and any interactions was not significant (Table 2). The highest germination for seeds in silicles and isolated seeds from autumn- and spring-germinating plants after 6 mo dry storage was 8% and 20%, respectively (Fig. 3). With an increase in cold stratification time, germination of seeds in silicles and isolated seeds from spring-germinating plants increased significantly at 4 °C during cold stratification (Fig. 3a) and at 5/2 °C after various periods of cold stratification (Fig. 3b). After 16 weeks at 4 °C or after 12 weeks at 4 °C plus 4 weeks at 5/2 °C, germination of seeds in silicles and isolated seeds was about 98% and 80%, respectively (Fig. 3). Although germination of seeds from autumn-germinating plants increased with increase in the cold stratification time, seeds in silicles and isolated seeds germinated to 50–60% during 12 weeks at 4 °C (Fig. 3a) and to 38–40% when transferred to 5/2 °C after 12 weeks of cold stratification at 4 °C (Fig. 3b). However, after 16 weeks of cold stratification at 4 °C, 98% of the seeds had germinated (Fig. 3a); thus, a germination test was not performed.

## Discussion

### Germination season causes flexibility of life history traits

Post-germination life history traits of *I. violascens* differed greatly between plants that germinated in autumn and spring. Thus, our first hypothesis that germination season would significantly affect the expression of plasticity in all major post-germination life history traits was confirmed. Further, autumn-germinating plants produced proportionally more seeds with intermediate PD than spring-germinating plants, while spring-germinating plants produced proportionally more seeds with nondeep PD than autumn-germinating plants. Thus, our second hypothesis that plants from autumn- and spring-germinating seeds (F_1_) would produce different proportions of seeds with nondeep and intermediate PD (F_2_) also was supported. Although many studies have tested the effects of germination season on plant life history traits such as survival, growth and reproduction^5^,^6^,^7^,^8^,^9^,^10^,^11^,^12^,^13^,^14^,^28^,^29^,^30^,^31^, ours is the first one to show that autumn- and spring-germinating plants differ in the proportion of seeds they produce with different levels of PD.

Dormancy/germination characteristics of seeds produced by plants from autumn- and spring-germinating seeds of *Thlaspi arvense* and *Capsella bursa-pastoris* may, or may not, differ. Germination percentages of seeds from overwintering plants of *T. arvense* did not differ significantly from those of spring-emerging plants^8^. GA_3_-treated seeds (to break dormancy) of spring-germinating plants of *T. arvense* germinated faster than those of autumn-germinating plants, but final germination percentage was the same. On the other hand, neither percentage nor rate of germination differed significantly for seeds from spring- and autumn-germinating plants of *C. bursa-pastoris.* However, an accelerated aging treatment (120 h at 42 °C) increased germination percentage of seeds of autumn-germinating more than that of spring-germinating plants of *C. bursa-pastoris*. Seeds of *T. arvense* from autumn-germinating plants lost viability during the accelerated aging test, while germination percentage of those from spring-germinating plants did not differ from that of the control.

Parental lines of *T. arvense* have been used to investigate effects of germination timing of mother plants on characteristics of the seeds they produce^32^. Seeds from autumn-germinating plants germinated to significantly higher percentages than those from spring germinators. This was true for parental lines in which the overwintering plants (winter annuals) or overwintering seeds (summer annuals) had been interrupted by a mid-winter warm period (simulating climatic warming or not (controls). In both summer and winter annuals, seeds from parental lines in which overwintering plants or seeds had not been exposed to mid-winter warming germinated to higher percentages than those that had been exposed to a mid-winter warming spell. Seeds from the winter annual lines were collected in late June and those from summer annual lines in late July. Seeds of all four parental lines were planted on 31 August.

The timing of germination has an effect on growth conditions encountered by seedlings and on survival. The germination behavior of *I. violascens* seeds allows them to germinate in autumn and in spring. Thus, autumn-germinating plants must have the ability to survive in a vegetative rosette state through drought in autumn and under the snow in winter, and both autumn- and spring-germinating plants must time flowering and allocate resources appropriately to flower and set seeds in spring before the onset of summer drought. Survival of spring germinants of *I. violascens* was higher than that of autumn germinants. In the weedy winter annual/ephemeral *Diplotaxis erucoides*^31^, survival varied with disturbance regimes and between and within cohorts^33^. Ninety-six percent of the rosettes (not watered) of both early- and late-autumn germinants of *Arabidopsis thaliana* survived the winter and bolted (initiation of reproduction), whereas no spring-germinating plants survived to this stage except those that received supplemental watering^9^. In *Diptychocarpus strictus*, a high percentage of seedlings of both autumn- and spring-germinating plants survived and reproduced^12^.

The timing of germination has an effect on seed production. The number of seeds produced per plant by autumn-germinators of *I. violascens* was higher than that produced by the spring germinators. Thus, autumn germination in *I. violascens* greatly increased this component of fitness of individuals in the population. Autumn-germinating plants of *Lactuca serriola* produced about 10 times more seeds per individual than spring- and summer-germinating plants^7^, and the number of seeds produced by autumn-germinating plants of *D. erucoides* was three to 10 times higher than that produced by spring germinators^31^,^33^. Compared to spring-germinating plants of *A. thaliana* grown in Rhode Island and Kentucky, USA, autumn-germinating plants produced a larger rosette, more basal branches and a greater total number of fruits, bolted at a later age and had a longer flowering interval^10^. Autumn-germinating plants of *D. strictus* produced about four times more seeds per individual than spring-germinating plants^12^. In *T. arvense*, on the other hand, summer annual (spring germinators) produced significantly more silicles and seeds than winter annuals (autumn-germinators)^8^,^32^.

Plant size, or biomass production, has an effect on allocation of resources to reproduction. The larger plants of *I. violascens* allocated the least proportion of biomass to reproduction and the smallest plants the greatest proportion. However, the absolute amount of biomass (g) increased with plant size. In *Teesdalia nudicaulis* (Brassicaceae), a later (early to late October) time of germination greatly decreased size of plant and seed production per plant but did not appreciably affect the number of seeds per fruit^5^. In the three South African desert ephemerals *Dimorphotheca sinuata*, *Ursinia calenduliflora* and *Heliophila pendula*^34^, and in the cold desert winter annual/spring ephemeral *D. strictus*^12^,^35^, total biomass allocated to reproduction increased with plant size. However, proportion of total biomass allocated to reproduction in these four species increased with decrease in plant size, which was related to time of germination and thus to length of growing season. Thus, like these four species plants of *I. violascens* that germinated in autumn were larger, produced more total reproductive biomass and allocated a smaller proportion of total biomass to reproduction than spring-germinating plants. In the two summer annual weeds *Amaranthus retroflexus* and *Chenopodium glaucum*, later-germinating plants were smaller, reproduced at an earlier age and allocated a greater proportion of biomass to reproduction than earlier-germinating plants^14^. Total biomass allocated to reproduction in the summer annual *Xanthium canadense* increased with plant size, whereas proportion of total biomass allocated to reproduction increased with decreased in plant size, which was related to germination season^36^.

Plant size and total number of fruits and seeds in *I. violascens* were significantly greater in autumn- than in spring-germinators, in agreement with other studies. Sans and Masalles^31^ reported that in the weedy winter annual/spring ephemeral *D. erucoides* reproductive biomass was positively (and linearly) correlated with vegetative biomass. Numerous studies on monocarpic plants have reported a reduction in seed production with delay in germination^5^,^6^,^7^,^11^,^12^.

The increase in proportion of biomass allocated to reproduction in *I. violascens* in a short growing season (autumn- versus spring-germinating plants) can be interpreted as a stress response. Likewise, stressful growth conditions caused a shift to a higher proportion of biomass to reproduction and increased the proportion of the “low risk” (i.e. low dispersal-high dormancy) lower indehiscent diaspores in *D. strictus*^35^. We interpret these plastic responses in life history traits to stress to be adaptive in that they increase the seed production component of fitness. For *I. violascens*, the negative effects of delaying germination until spring (short season for maximum growth) are reduced by the ability of plants to allocate a high proportion of resources to reproduction under these stressful conditions.

### Seed dormancy exhibits transgenerational plasticity

Differences in seed dormancy may be due to genetics, parental environment (including epigenetics) or genetics × parental environment interactions^1^,^27^. Differences in dormancy of seeds from autumn- and spring-germinating plants seeds are not due to genetics because autumn- and spring-germinating plants produced seeds with both levels of PD. However, many factors of the parental plant environment can have nongenetic transgenerational effects on dormancy/germination and on other features of growth and functioning of the F_1_ progeny and in some cases of the F_2_ progeny and beyond^1^,^27^,^37^,^38^,^39^,^40^,^41^. In general, it seems that the post-zygotic parental plant growth environment, and not the prezygotic environment, affects the degree of seed dormancy^42^,^43^,^44^. However, the pre-zygotic parental plant environment can affect seed dormancy^45^,^46^,^47^ and also seed longevity^48^,^49^. In *I. violascens*, the post-zygotic environment of autumn- versus spring-germinating plants did not differ. The period from opening of the first flower to maturation of the last silicle was from 5 May to 8 June 2014 for autumn-germinating plants, when mean daily maximum/minimum temperatures were 23.4/12.3 °C, and from 9 May to 6 June 2014 for spring-germinating plants, when mean daily maximum/minimum temperatures were 23.2/12.0 °C. Moreover, rainfall during the seed maturation period of autumn- and spring-germinating plants was the same (i.e. 32.5 mm). In contrast, the pre-zygotic environment of autumn- and spring-germinating plants differed greatly. The period from seedling emergence to appearance of the first flower bud was 1 October 2013 to 28 April 2014 for autumn-germinating plants and 24 March to 2 May 2014 for spring-germinating plants, when mean daily maximum/minimum temperatures were 3.4/−4.9 °C and 15.9/5.5 °C, respectively, and rainfall 194.4 mm and 83.4 mm, respectively.

The seed stage can influence subsequent plant phenology, e.g. through differences in germination timing^9^,^11^,^12^ and also through physiological processes independent of germination timing^50^. Thus, it seems reasonable to think that differences in the F_2_ of autumn- and spring-germinating plants could, at least in part, be related to differences in the environment of the seeds from which those two cohorts were produced. That is, the seeds that gave rise to autumn-germinating plants were exposed to high summer temperatures and germinated in autumn, while seeds from spring-germinating plants were exposed to high summer followed by low winter temperatures and then germinated in spring.

One way in which nongenetic transgenerational plasticity may be mediated is by changes in maternal provisioning to the seed of storage reserves (seed mass) and in seed coat structure and thickness^26^,^27^,^38^, which can affect dormancy and thus timing of germination and various post-germination plant growth and life history traits of offspring. However, maternal provisioning does not explain the differences in dormancy and germination of seeds produced by autumn- and spring-germinating plants of *I. violascens*. The oven-dry mass (mg) (five replications of five each) of autumn- and spring-germinating plants’ silicles, seeds, embryos, pericarps and seed coats were: silicles 39.56 ± 0.7 and 39.08 ± 0.8, respectively; seeds 20.14 ± 1.86 and 19.82 ± 9.12, respectively; embryos 18.28 ± 0.47 and 18.26 ± 0.39, respectively; pericarps 19.26 ± 6.47 and 19.26 ± 1.53, respectively; and seed coats 2.04 ± 0.08 and 1.96 ± 0.07, respectively. None of these five comparisons differed significantly (t-test, P > 0.05) (Lu *et al.*, unpublished). The endosperm in mature seeds of Brassicaceae is very thin^51^ and thus would not represent significant differences in seed provisioning by the mother plant to autumn- and spring-germinating plants.

A second way in which nongenetic transgeneration effects can be mediated is through epigenetics^52^,^53^,^54^. In epigenetics, gene expression is modified (change in gene activity) by abiotic and biotic environmental stress through molecular mechanisms (other than DNA sequence change), such as (cytosine) DNA methylation (5′meC) and histone modification (epigenetic marks) directed and maintained by small RNA molecules (epigenetic mechanism or epigenetic regulation). Cold stress, which is known to cause transgenerational effects in plants^27^, on the overwintering rosettes of autumn-germinating plants is one of the big pre-zygotic environmental differences between autumn- and spring-germinating plants and possibly helps to explain why seeds from autumn-germinating plants were more dormant than those from spring-germinating plants.

Nongenetic transgenerational effects can be adaptive when the offspring grow in (not dispersed away from) their mother’s environment and the environment is predictable between generations^55^,^56^,^57^,^58^. That is to say, offspring responses may be adaptive to the same environmental factor (or cue), e.g. cold stress, that triggered the response in the parental or ancestral generation. If cold stress on the rosette did cause autumn-germinating plants to produce more seeds that require cold stratification to germinate compared to spring-germinating plants, then *I. violascens* may be an example of this kind of transgenerational response.

In any case, it is clear that the environment during the growth/juvenile stage of the life cycle of *I. violascens*, and not that during the seed maturation stage, has an effect on the proportions of seeds with nondeep and intermediate PD that are produced. Further, variation in post-germination traits could influence natural selection^9^,^59^. In *I. violascens*, the timing of germination (autumn- versus spring-germinating plants) not only results in different morphological phenotypes but also in different levels of fitness and variation in proportion of seeds with nondeep and intermediate PD. Thus, the capacity for both spring and autumn germination of *I. violascens* seeds means that there is much variation in this species that could be subjected to natural selection.

The variation in the proportions of seeds with nondeep and intermediate PD is of special interest in terms of future climate change. Increase in amount of rainfall in autumn would increase the number of seeds of *I. violascens* that could germinate in autumn, but as our results show autumn-germinating plants produce seeds with both nondeep and intermediate PD. On the other hand, less rain in autumn would increase the number of seeds that germinate in spring. However, spring-germinating plants also produce seeds with both levels of PD. Thus, from a seed dormancy/germination perspective, natural selection in *I. violascens* has resulted in a mechanism (i.e. production of two levels of PD) that ensures the potential for some seeds from both autumn- and spring-germinating plants to germinate in autumn and in spring.

## Methods

### Seed collection

Freshly-matured fruits (silicles) were collected from dry infructescences of several hundred *I. violascens* plants growing on a cold desert sand dune in Fukang city in the southern part of the Junggar Basin of Xinjiang Province (44°22′N, 88°08′E, 458 m a.s.l.), China. This area of the Junggar Basin has a temperate continental climate. Mean annual temperature is 7.9 °C, and mean temperature of the coldest (January) and hottest (July) month is −17.0 °C and 26.0 °C, respectively. Average annual precipitation (including rain and snow) is 202.2 mm, about two-thirds of which falls in spring and summer. The snow that falls in winter begins to melt in March or April (data from Fukang weather station, 2001–2013). Annual potential evaporation is >2000 mm^60^. Seeds were stored in paper bags at room conditions (16–30 °C, 10–40% RH) until used.

### Effect of germination season on flexibility of life history traits

**Figure 4:**
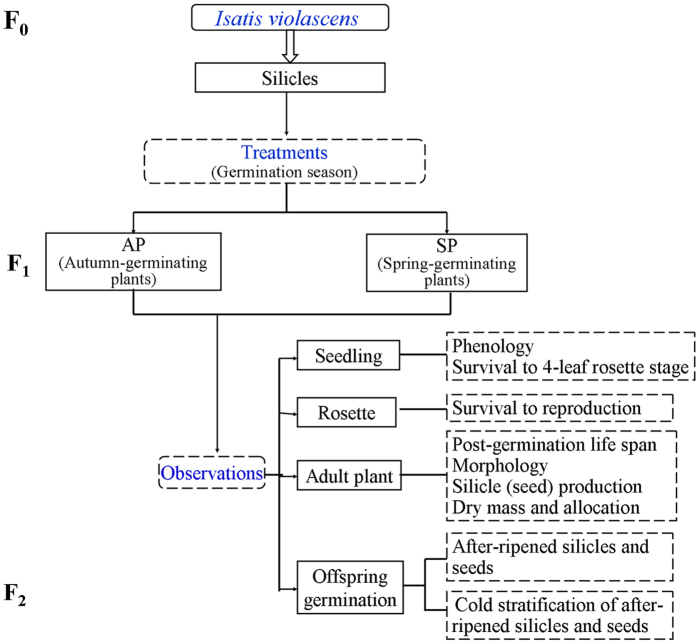
Experimental design of study on *Isatis violascens*.

On 24 August 2013, 2000 seeds isolated from silicles were sown on bare soil in plots (2.0 m × 1.5 m) in the experimental garden located on the campus of Xinjiang Agricultural University, Urümqi, China, in the southernmost part of the Junggar Desert. The soil was watered to field capacity every 3 days to ensure that water was not a limiting factor for germination or for growth of the resulting plants. Plots were not watered during winter when the soil was frozen. At 7-day intervals from August 2013 to June 2014, germinated seeds (seedlings) were counted and marked. Information on temperature, rainfall and snowfall at the study site (Urumqi) for 2013 and 2014 was obtained from data collected at the National Meteorological Information Center, China Meteorological Administration. The overall experimental design is shown in Fig. 4.

A total of 93 plants (31 autumn-germinating plants + 62 spring-germinating plants) was used to determine seedling survival. Also, 46 surviving seedlings (16 autumn-germinating plants + 30 spring-germinating plants) were marked in order to monitor several other life history traits, including phenology, morphological characters, silicle (seed) production and dry mass accumulation and allocation.

#### Phenology

Emergence date (number of days since sowing until all seeds in each treatment had emerged), flowering date (number of days since sowing until all plants in each treatment had flowered), fruiting date (number of days since sowing until occurrence of the first green fruit on all plants in each treatment) and maturation date (number of days since sowing until the first fruit of all individuals in each treatment had turned a khaki-color and were ready to disperse naturally) were determined. Then, growth period (interval from emergence to final height), flowering period (interval from first flower that bloomed to last flower that withered in each treatment), fruiting period (interval from appearance of the first fruit to maturation of last fruit in each treatment) and post-germination life span (interval from emergence of the first individual to death of the last individual in each treatment) were determined.

#### Survivorship

The number of seedlings surviving from germination to the four-leaf rosette stage and the number of plants surviving from this stage to reproduction were recorded.

#### Morphological characters

Plant height (H, from soil surface to highest apical meristem) and number of branches (BN, >1 cm in length on main stem) were determined. The average height growth rate per week (H) over the post-germination life span was calculated using the formula H = h/l (cm·d^−1^), where h is plant height at maturity and l life period. The diameter of each rosette also was recorded on the day the seedlings reached the four-leaf stage, which is the beginning of the rosette stage.

#### Silicle (seed) production

At harvest, number of infructescences per individual and of silicles per individual was determined.

#### Dry mass accumulation and allocation

Mature autumn- and spring-germinating plants were harvested and separated into vegetative (root, stem and leaves) and reproductive (silicles, i.e. including their pericarps and seeds) organs. Then, all parts were oven-dried at 80 °C for 48 h and weighed using a Sartorius BS210S electronic-balance (0.0001 g). Total biomass is vegetative plus reproductive biomass. Allocation to roots, stems, leaves and reproductive organs was expressed as a percentage of the total dry mass.

### Transgenerational plasticity in seed dormancy

#### Seed collection

Freshly-matured khaki-colored silicles that were dispersing naturally were collected from the autumn- and spring-germinating plants in early June 2014, separated into silicles from autumn- and spring-germinating plants and stored in paper bags at room conditions (16–30 °C, 10–40% RH) to allow them to afterripen.

#### Presence of nondeep PD

By conducting tests for presence of nondeep and intermediate PD in seeds from both autumn- and spring-germinating plants, we can determine if the dormancy ratio (nondeep: intermediate) varied when plants had different life histories. If seeds of *I. violascens* have nondeep PD, they will afterripen during dry storage at room temperature^22^. After silicles from autumn- and spring-germinating plants had been stored dry in the laboratory for 0 and 6 months (i.e. until 15 December 2014), intact silicles and isolated seeds from autumn- and spring-germinating plants were incubated in Petri dishes on wet filter paper at optimum conditions for germination (5/2 °C, in darkness)^22^. Four replicates of 25 intact silicles and of 25 isolated seeds each of autumn- and spring-germinating plants were used to test germination. Silicles and seeds were checked only after 28 d, and germination percentages were calculated based on number of viable seeds.

#### Presence of intermediate PD

Seeds of *I. violascens* with intermediate PD will germinate after they have been dry stored (afterripened) for 6 months and then given a cold stratification treatment^22^. To determine the proportion of seeds from autumn- and spring-germinating plants that had intermediate PD, we used seeds from both kinds of plants that had been stored dry at room temperature for 6 months. Six-month-old intact silicles and isolated seeds from autumn- and spring-germinating plants were cold stratified on moist filter paper at 4 °C for 0, 4, 8, 12 and 16 weeks. After each period of cold stratification, four replications of 50 silicles and of 50 seeds were checked for germination. Then, four replicates of 25 intact silicles and of 25 isolated seeds, which did not germinate after each period of cold stratification, were tested for germination as described above. However, 98% of the seeds germinated during the 16 weeks of cold stratification, and thus no germination test per se was performed.

### Statistical analysis

To identify the main traits of life history tested, a principal component analysis (PCA) model was initially used to determine principal components with eigenvalues >1 for life history traits (i.e. phenology, morphological characters, silicle production and dry mass accumulation and allocation). Then, a one-way ANCOVA was used to test for significant differences between the two germination seasons (autumn- and spring-germinating plants) for the above principal components. Germination season was considered a fixed effect. Also, seedling size (i.e. diameter at four-leaf rosette stage) was used as a covariate to minimize the effects of variation in initial size among seedlings on dependent variables in these analyses. Correlative analyses were used to determine the relationship between dry mass of reproductive organs and plant height and between dry mass allocation to reproductive organs and plant height.

Germination data were analyzed using generalized linear models (GLMs) with a logit link to germination as a binomial response variable (two categories: germinated versus non-germinated). In the models, germination timing of mother plants (autumn- and spring-germinating plants), treatment (intact silicles and isolated seeds) and storage time (0 and 6 months) were used as fixed factors for the “Presence of nondeep PD” experiment, and germination timing of mother plants, treatment and cold stratification time (0, 4, 8 and 12 weeks) were used as fixed factors for the “Presence of intermediate PD” experiment, with their interaction included in models. The significance of effects of fixed factors and their interactions in the models was tested by Wald χ^2^ values. Tukey’s HSD test was performed for multiple comparisons to determine significant differences among treatments. Statistical tests were conducted at P = 0.05. All data analyses were performed with the software SPSS 16.0 (SPSS Inc, Chicago, Illinois, USA). Values are means ± 1 s.e. (i.e. standard errors).

## Additional Information

**How to cite this article**: Lu, J. J. *et al.* Effects of germination season on life history traits and on transgenerational plasticity in seed dormancy in a cold desert annual. *Sci. Rep.* **6**, 25076; doi: 10.1038/srep25076 (2016).

## Supplementary Material

Supplementary Tables

## Figures and Tables

**Table 1:** Effect of germination season/watering regime on phenology, dry mass of plants and dry mass allocation to silicles in *Isatis violascens* (mean ± 1 s.e.).

	Autumn-germinating plants	Spring-germinating plants	F-value	P-value
Phenology				
Flowering date (d)	246.81 ± 1.76	250.73 ± 2.47	3.59	0.07
Fruiting date (d)	254.31 ± 2.14	257.57 ± 2.44	0.66	0.42
Post-germination life span (l, d)	231.13 ± 1.88	70.97 ± 0.51	1989.60	<0.05
Dry mass of plants	4.12 ± 0.17	0.70 ± 0.03	106.57	<0.05
Dry mass allocation to silicles	32.56 ± 2.10	55.72 ± 1.51	16.333	<0.05

A one-way ANCOVA was used to test significant differences between autumn- and spring-germinating plants. l, post-germination life period. Dates for flowering and fruiting are number of days (d) since sowing and post-germination life span interval from emergence to death.

**Table 2:** Generalized linear models of effects of germination timing of mother plants (M), treatment (T), storage time (S) and their interactions on presence of nondeep PD and of germination timing of mother plants (M), treatment (T), cold stratification time (C) and their interaction on presence of intermediate PD in *Isatis violascens*.

Factor	d.f	χ^2^	P-value
Presence of nondeep PD			
M	1	7.671	<0.05
T	1	0.827	<0.05
S	1	14.228	0.36
M × T	1	0.360	<0.05
M × S	1	0.066	0.55
T × S	1	2.567	0.80
M × T × S	1	0.562	0.11
Presence of intermediate PD			
M	1	157.02	<0.05
T	1	3.02	0.08
C	3	171.07	<0.05
M × T	1	0.06	0.80
M × C	3	3.38	0.34
T × C	3	0.20	0.98
M × T × C	3	0.37	0.95
