# Supplementary material for: Effects of germination season on life history traits and on transgenerational plasticity in seed dormancy in a cold desert annual
Source: Sci Rep. 2016 Apr 27;6:25076. doi: 10.1038/srep25076 (PMC4846867; doi:10.1038/srep25076)
Supplement: Supplementary Tables [file srep25076-s1.pdf]

Effects of germination season on life history traits and on transgenerational plasticity in seed dormancy in a cold desert annual

Juan J. Lu[1], Dun Y. Tan[1], Carol C. Baskin[1, 2, 3] & Jerry M. Baskin[1, 2]

Supplementary Table S1. Effect of germination season/watering regime on phenology, morphology and silicle production of plants in *Isatis violascens* (mean ± 1 s.e.). A one-way ANCOVA was used to test significant differences between autumn- and spring-germinating plants. H, plant height at maturity; l, post-germination life period. Dates for emergence, flowering and fruiting are number of days (d) since sowing; flowering interval, interval from blooming of first flower to withering of last flower; and post-germination life span, interval from emergence to death.

	Autumn-germinating plants	Spring-germinating plants
Phenology		
Emergence date (d)	37.75 ± 0.54[a]	211.53 ± 1.01[b]
Flowering date (d)	246.81 ± 1.76[a]	250.73 ± 2.47[a]
Fruiting date (d)	254.31 ± 2.14[a]	257.57 ± 2.44[a]
Flowering period (d)	39.00 ± 0.97[b]	33.00 ± 0.33[a]
Post-germination life span (l, d)	231.13 ± 1.88[b]	70.97 ± 0.51[a]
Morphology		
Plant height at maturity (H, cm)	66.18 ± 1.82[b]	49.22 ± 1.23[a]
Silicle production per individual		
Number of infructescences	21.69 ± 2.80[b]	4.87 ± 0.63[a]
Number of silicles	174.38 ± 11.35[b]	51.97 ± 3.87[a]

Supplementary Table S2. Pearson correlations among characters for AP and SP plants in *Isatis violascens* . N = 180. * $P < 0.05$; ** $P < 0.01$; *** $P < 0.001$.

	Emergence date	Flowering date	Fruiting date	Maturation date	Flowering period	Fruiting period	Growth period	Post-germination life span	Plant height at maturity	Number of branches	Height growth rate per week	Number of infructescenses	Number of silicles per individual	Dry mass of root	Dry mass of stem	Dry mass of leaf	Dry mass of silicle	Dry mass of plant	Dry mass allocation to root	Dry mass allocation to stem	Dry mass allocation to leaf	Dry mass allocation to silicle
Emergence date	1.00																					
Flowering date	0.14	1.00																				
Fruiting date	0.15	0.62***	1.00																			
Maturation date	0.07	0.52***	0.90***	1.00																		
Flowering period	-0.70***	-0.1	-0.1	-0.05	1.00																	
Fruiting period	-0.52***	-0.26	-0.30*	-0.31*	0.59***	1.00																
Growth period	-0.81***	0.33*	0.17	0.2	0.66***	0.40**	1.00															
Post-germination life span	-0.57***	-0.14	-0.23	-0.14	0.59***	0.53***	0.65***	1.00														
Plant height at maturity	-0.59***	-0.13	-0.16	-0.05	0.52***	0.24	0.64***	0.54***	1.00													
Number of branches	-0.73***	-0.15	-0.15	-0.06	0.62***	0.47**	0.75***	0.77***	0.75***	1.00												
Height growth rate per week	0.65***	0.15	0.15	0.1	-0.59***	-0.59***	-0.66***	-0.83***	-0.24	-0.65***	1.00											
Number of infructescenses	-0.69***	-0.22	-0.19	-0.07	0.58***	0.45**	0.67***	0.61***	0.74***	0.80***	-0.55***	1.00										
Number of silicles per individual	-0.59***	-0.16	-0.09	-0.04	0.51***	0.39**	0.65***	0.69***	0.71***	0.77***	-0.60***	0.66***	1.00									
Dry mass of root	-0.75***	-0.15	-0.07	0.04	0.62***	0.48**	0.73***	0.62***	0.74***	0.73***	-0.56***	0.73***	0.75***	1.00								
Dry mass of stem	-0.66***	-0.17	-0.13	-0.08	0.60***	0.53***	0.70***	0.81***	0.69***	0.85***	-0.67***	0.73***	0.76***	0.76***	1.00							
Dry mass of leaf	-0.77***	-0.19	-0.21	-0.15	0.68***	0.52***	0.71***	0.64***	0.58***	0.73***	-0.67***	0.63***	0.60***	0.68***	0.66***	1.00						
Dry mass of silicle	-0.60***	-0.13	-0.06	-0.02	0.53***	0.40**	0.67***	0.67***	0.68***	0.78***	-0.61***	0.68***	0.96***	0.78***	0.77***	0.58***	1.00					
Dry mass of plant	-0.66***	-0.14	-0.12	-0.05	0.58***	0.47**	0.70***	0.67***	0.74***	0.82***	-0.58***	0.75***	0.91***	0.85***	0.85***	0.69***	0.95***	1.00				
Dry mass allocation to root	-0.2	0	0.19	0.25	0.08	0.08	0.15	-0.04	0.26	0	0.1	0.17	0.02	0.55***	0.14	0.01	0.05	0.14	1.00			
Dry mass allocation to stem	-0.23	-0.17	-0.07	-0.03	0.22	0.29	0.23	0.46**	0.25	0.34*	-0.33*	0.29*	0.11	0.27	0.59***	0.14	0.08	0.17	0.25	1.00		
Dry mass allocation to leaf	-0.69***	-0.26	-0.32*	-0.23	0.61***	0.48**	0.58***	0.54***	0.44**	0.59***	-0.65***	0.57***	0.39**	0.50***	0.47**	0.90***	0.34*	0.44**	-0.08	0.13	1.00	
Dry mass allocation to silicle	0.76***	0.18	0.21	0.12	-0.65***	-0.54***	-0.68***	-0.63***	-0.50***	-0.68***	0.68***	-0.63***	-0.37*	-0.61***	-0.65***	-0.87***	-0.34*	-0.49**	-0.12	-0.43**	-0.92***	1.00
